# Serious Non-AIDS events: Immunopathogenesis and interventional strategies

**DOI:** 10.1186/1742-6405-10-29

**Published:** 2013-12-13

**Authors:** Denise C Hsu, Irini Sereti, Jintanat Ananworanich

**Affiliations:** 1 The Kirby Institute for Infection and Immunity in Society, University of New South Wales, Sydney, Australia; 2 HIV Pathogenesis Unit, Laboratory of Immunoregulation, NIAID/NIH, Bethesda, MD, USA; 3 HIV Netherlands Australia Thailand Research Collaboration and SEARCH, Thai Red Cross AIDS Research Centre, Bangkok, Thailand

**Keywords:** Serious non-AIDS events, Immune activation, HIV infection

## Abstract

Despite the major advances in the management of HIV infection, HIV-infected patients still have greater morbidity and mortality than the general population. Serious non-AIDS events (SNAEs), including non-AIDS malignancies, cardiovascular events, renal and hepatic disease, bone disorders and neurocognitive impairment, have become the major causes of morbidity and mortality in the antiretroviral therapy (ART) era. SNAEs occur at the rate of 1 to 2 per 100 person-years of follow-up. The pathogenesis of SNAEs is multifactorial and includes the direct effect of HIV and associated immunodeficiency, underlying co-infections and co-morbidities, immune activation with associated inflammation and coagulopathy as well as ART toxicities. A number of novel strategies such as ART intensification, treatment of co-infection, the use of anti-inflammatory drugs and agents that reduce microbial translocation are currently being examined for their potential effects in reducing immune activation and SNAEs. However, currently, initiation of ART before advanced immunodeficiency, smoking cessation, optimisation of cardiovascular risk factors and treatment of HCV infection are most strongly linked with reduced risk of SNAEs or mortality. Clinicians should therefore focus their attention on addressing these issues prior to the availability of further data.

## Introduction

Since the first description of AIDS in 1981, there have been tremendous advances in understanding the biology of the virus, the host’s immune response and the clinical management of HIV infection. The introduction of combination antiretroviral therapy (ART) in 1996 has revolutionized HIV treatment, increasing the average life expectancy after HIV diagnosis from 10.5 to 22.5 years from 1996 to 2005 [1]. The estimated life expectancy for a 30 year old male infected with a drug-sensitive virus in 2010 and starting ART at about 6 years post infection can be as high as 75 years in some predictive models [2].

Despite the success of ART, life expectancy in HIV-infected patients is still lower than uninfected persons [2-4] and mortality in HIV-infected patients can be up to 15 times higher when compared with the general population, matched for sex and age [3]. In the pre-ART era, AIDS was the primary cause of death in HIV-infected patients [5-7]. With the use of ART, mortality due to serious non-AIDS events (SNAEs) has become more prominent especially in resource-rich settings [6,8-13] and in patients with higher CD4 T cell counts [7,14].

## Definition of serious non-AIDS events

Non-AIDS events (NAEs) are clinical events that do not meet the definition of AIDS-defining events based on the 1993 US Centers for Disease Control and Prevention (CDC) AIDS indicator conditions [15]. They encompass multiple diseases involving different organ systems, including cardiovascular, liver and renal disease, non-AIDS-defining malignancies, diabetes, neuropsychiatric disorders and bone-related abnormalities [16].

SNAEs are NAEs that result in death, are life-threatening, cause prolonged hospitalization and persistent incapacity or are associated with significant morbidity [12,14,17]. Most studies include cardiovascular, liver and end stage renal disease, as well as non-AIDS-defining cancers [11,14,18,19]. Other studies include an even broader range of conditions such as non-AIDS-related infections and psychiatric events [7,12,16,17,20].

## Incidence of SNAEs

The incidence of SNAEs in ART-treated patients is around 1 to 2 per 100 person-years of follow-up (PYFU) [11,14,17-19,21], (Table 1), but can be up to 60 per 100 PYFU in a cohort of treatment-experienced patients with multidrug resistant virus [12]. The relative contribution of non-AIDS malignancy, cardiovascular, liver and end stage renal disease to SNAEs vary across studies due to inconsistencies in the definition of SNAEs and differences in the rates of underlying co-morbidities e.g. Hepatitis B virus (HBV) and Hepatitis C virus (HCV) co-infection. However, non-AIDS malignancy, cardiovascular disease (CVD) and liver disease combined seem to account for >80% of SNAEs according to several published studies [9,11,14,17,18]. The incidence of non-AIDS malignancy and cardiovascular disease is about 2-fold higher in HIV-infected patients in the ART era when compared to the general population [22-26].

**Table 1:** Summary of studies describing the incidence of SNAEs in various patient populations

**Study**	**Study population**	**N**	**Median follow-up (yrs)**	**Male (%)**	**Median age (yrs)**	**Median nadir CD4 count (cells/μL)**	**Median baseline CD4 count (cells/μL)**	**HBV + (%)**	**HCV + (%)**	**Rate of SNAEs per 100 PYFU**	**Ref**
EuroSIDA	A prospective observational cohort of HIV-infected patients in Europe, Israel and Argentina followed from 2001-09.	12844		73	39	178	403	6	24	1.8	[14]
SMART (S) ESPRIT(E)	S: HIV-infected patients with CD4 count >350 cells/μL were randomized to either CD4 count guided episodic use of ART or to continuous use of ART. E: HIV-infected patients with CD4 count >300 cells/μL were randomized to interleukin-2 plus ART or to ART alone.	S: 5472 E: 4111	S: 2.4 E: 6.8	S: 73 E: 81	S: 43 E: 40	S: 250 E: 197	S: 597 E: 457	S: 2	S: 15	1	[11,27,127]
	An observational cohort of HIV-infected patients with CD4 count >500 cells/μL in Spain from 1996-2011.	547	10	80	43	348	630	5	28	1.4	[17]
CoRIS	A prospective multicenter observational cohort of HIV-infected patients in Spain followed from 2004-2010.	5185	2.1	79	36		342	4	12	2.9	[16]
	A retrospective study of HIV-infected patients receiving ART in Botswana (B) and Nashville, USA (US); from 2002 (B) and 2003 (US)-2007.	B: 650 US: 1129	B: 3 US: 1.5	B: 31 US: 74	B: 33 US: 40		B: 199 US: 243			B: 1 US: 1.2	[19]
LATINA	A retrospective study of HIV-infected patients in Latin America from 1997-2007.	6007	2.5	70						0.9	[18]
APROCO/COPILOTE	A prospective observational cohort of HIV-infected patients in France followed from 1997-2006.	1231	7.3	77	36		279	5	23	10.5	[20]
OPTIMA	HIV-infected patients with resistance to at least 2 different multidrug regimens were randomized to (a) re-treatment with either standard (≤4) or intensive (≥5) antiretroviral drugs and (b) either treatment starting immediately or after a 12-week monitored ART interruption.	368	4	98	48		107	11	22	61.0	[12]
ATHENA	An observational cohort of ART naive HIV-infected patients starting ART in the Netherlands, 1996-2010.	6440	3.9	75	39		200	7	6	1.2	[21]

SNAEs are associated with worse outcome than AIDS events in the ART era. Compared with ART-treated patients without events, the risk of death is increased by 7 to 11-fold in those with SNAEs and by 4 to 5-fold in those with AIDS events [11,14]. Amongst SNAEs, liver-related events are associated with the highest mortality [11,14], followed by renal events, malignancies and cardiovascular events, with estimated 12-month mortality of 39.7, 32.7, 29.5 and 6.1% respectively [11].

## SNAEs pathogenesis

Prior to the Strategies for Management of Antiretroviral Therapy (SMART) Study, ART toxicities were thought to be a major contributor to SNAEs. In the SMART Study, over 5000 HIV-infected patients with CD4 T cell count >350 cells/μL were randomized to either episodic ART (when CD4 T cell count fell below 250 cells/μL) or continuous ART. Patients on episodic ART had 1.8-fold increase in mortality and 1.7-fold increase in SNAEs (defined as major cardiovascular, renal or hepatic disease) when compared to those on continuous ART [27], thereby highlighting the role of HIV viraemia and immunodeficiency in the pathogenesis of SNAEs [28].

**Figure 1:**
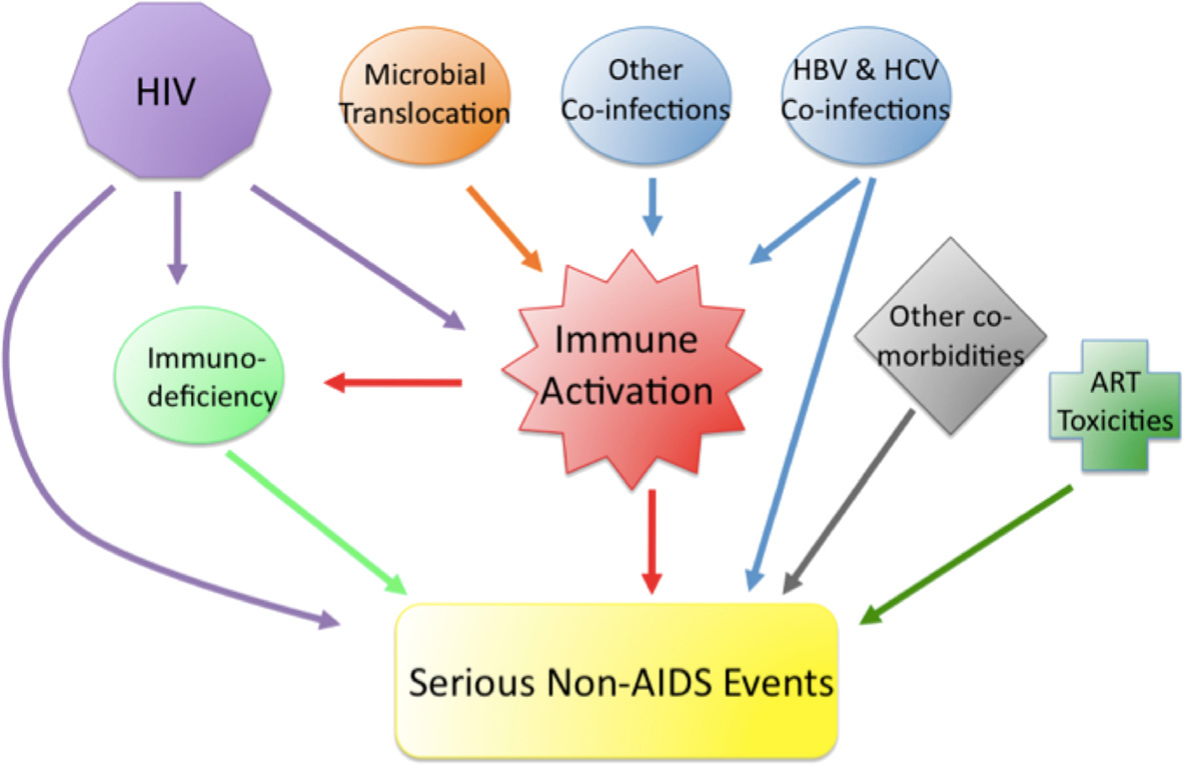
**Pathogenesis of serious non-AIDS events.** HIV infection causes progressive decline in CD4 T cells through direct cytopathic effects and immune mediated killing of infected cells, as well as indirectly via immune activation. Other drivers of immune activation include co-infections and microbial translocation. HIV can contribute to organ dysfunction through detrimental effects on hepatic stellate cells and renal tubular cells. HIV may also be oncogenic. Co-infection with HBV and HCV is especially important in liver related events. In addition, patients’ underlying co-morbidities e.g. smoking, cardiovascular risk factors, and ART related toxicities also contribute to SNAEs.

The pathogenesis of SNAEs is in fact multifactorial and complex (Figure 1). The direct effect of HIV and associated immunodeficiency, underlying co-morbidities and co-infections, immune activation with associated inflammation and coagulopathy as well as ART toxicities can all contribute.

### The direct effect of HIV

Uncontrolled HIV replication causes immune activation and progressive decline in CD4 T cell count [29]. In addition, HIV can also contribute directly to organ dysfunction and SNAEs. HIV can infect human hepatic stellate cells and induce collagen expression and pro-inflammatory cytokines secretion *in vitro*[30,31]. HIV can also mediate dysregulation of glomerular podocytes in HIV-transgenic mouse models [32], as well as apoptosis of human renal tubular cell lines [33]. Therefore, direct effect of HIV may contribute to decline in renal function and increase risk of chronic kidney disease [34]. HIV may contribute directly to non-AIDS malignancy [35], as it can be oncogenic by activating proto-oncogenes [36] or by blocking tumour suppressor genes [37] in cell lines. Increased rates of microsatellite instability in tumours of HIV-infected patients have also been found [38].

### Immunodeficiency

Lower nadir or pre-ART CD4 T cell count is associated with increased risk of SNAEs [11,14,16,39]. In addition, the degree of CD4 T cell recovery after ART initiation also influences the incidence of SNAEs [14,18,21,39]. A 100 cells/μL lower latest CD4 T cell count in ART-treated patients is associated with a 30% increase in the risk of SNAEs, even after adjusting for smoking status, diabetes mellitus, hyperlipidaemia, HCV and HBV co-infection and alcohol abuse [18]. Lower latest CD4 T cell count in ART-treated patients is also associated with an increase in mortality [40,41].

Suboptimal restoration of CD4 T cells may be secondary to a number of factors including decreased thymic function [42,43] and impaired homeostatic responses and survival of T cells [44]. HIV replication and immune activation stimulate the secretion of transforming growth factor (TGF-β), mainly by regulatory T cells, with macrophages also contributing. TGF-β triggers collagen production by fibroblasts [45-47]. The resultant structural damage and fibrosis of the lymphoid tissues restricts T cell access to interleukin-7 (IL-7) on the fibroblastic reticular cell (FRC) network [45] thus limiting naïve CD4 T cell survival [48,49]. Furthermore, ongoing immune activation leads to rapid CD4 T cell turnover, overwhelming the already impaired renewal mechanisms [44], resulting in suboptimal CD4 recovery [50,51].

### Co-infections

Due to common routes of transmission, HIV-infected patients also have increased risk of exposure to other infections that can cause organ dysfunction.

About 6-14% of HIV-infected patients have HBV and about 25-30% have HCV infection, with the rates varying depending on endemicity of HBV in the population and the prevalence of injecting drug use [52]. HBV and/or HCV co-infection greatly increases the risk of SNAEs despite ART [9,11,14,18,39,53]. Co-infection with HCV is associated with increased risk of renal disease (1.5-fold) [54,55], cardiovascular disease (1.5-fold) [56], cirrhosis (19-fold) and hepatocellular carcinoma (5-fold) [57] when compared with HIV mono-infected patients in the ART era.

HIV-infected patients also have increased risk of exposure to Human papillomavirus (HPV) [58], that is implicated in the pathogenesis of cervical and anal cancer [59,60].

### Other co-morbidities

HIV-infected patients also have higher prevalence of traditional cardiovascular risk factors such as smoking [61-63], elevated total cholesterol/HDL ratio [61-64] and substance abuse [65]. Some studies also found higher rates of hypertension and diabetes [64,66], but these findings have not been confirmed by others [62,63,67].

Smoking is a major cause of increased mortality in ART-treated patients, accounting for a loss of >12 life years, and is associated with >4-fold increase in all-cause mortality, >5-fold increase in non-AIDS mortality, >4-fold increase in cardiovascular-related mortality and >3-fold increase in cancer-related mortality [68].

Though uncontrolled HIV replication, immunodeficiency, co-infection and co-morbid conditions are important contributors to the pathogenesis of SNAEs, these factors only partially account for the increased risk of SNAEs in HIV-infected patients. HIV elite controllers have increased coronary plaques and carotid intima media thickness when compared to uninfected controls even after adjustment for traditional cardiovascular risk factors [69,70], demonstrating that factors other than uncontrolled viral replication, immunodeficiency, traditional risk factors, and ART toxicities contribute to increased cardiovascular risks in HIV-infected patients.

### Immune activation

Inflammation is central to the process of atherosclerosis [71,72], tumour progression [73,74] and liver fibrosis [75,76] in the general population. High levels of biomarkers associated with inflammation (C-reactive protein, CRP, and IL-6) and coagulopathy (D-dimer and fibrinogen) are associated with increased risk of cardiovascular disease [77-80], increased cancer risks [81-83] and mortality [80,84-86] in the general population.

In HIV-infected patients, CRP, IL-6 and D-dimer levels [61] as well as markers of T cell activation [50,87] remain higher than uninfected controls despite suppressive ART. Furthermore, higher CRP, IL-6 and D-dimer [88-90], soluble CD14 (sCD14, a marker of LPS-induced monocyte activation) [91] and lymphocyte activation markers [92] are associated with higher mortality.

In ART-treated patients, CRP, IL-6 and D-dimer levels are also associated with increased risk of CVD, independent of other CVD risk factors [93,94]. These markers are also associated with increased risks of both infection-related and infection unrelated cancers even after adjusting for demographics and CD4 T cell count [95]. Recently, higher levels of tumour necrosis factor (TNF) were also found to be significantly associated with increased risk of SNAEs [96]. Therefore these data suggest that immune activation plays a very important role in SNAEs pathogenesis.

#### ***Intermittent viraemia***

The drivers of immune activation are diverse. Intermittent HIV viraemia can occur in 20-30% of ART-treated patients [97,98]. The presence of viraemia whilst on ART is associated with higher IL-6, D-dimer and sCD14 levels [62] and SNAEs [11,39]. In addition, viraemia below the limit of detection of conventional assays also correlates with persistent T cell activation [99]. Therefore, residual viraemia may partially be responsible for persistent immune activation despite ART.

#### ***Co-infections***

The presence of co-infections also contributes to continual stimulation and activation of the immune system. Asymptomatic CMV infection has been associated with CD8 T cell activation in ART-treated patients [100]. CMV specific CD4 T cells can cause a systemic inflammatory response that is sustained even during latent infection [101] and is associated with atherogenesis [102,103]. Hepatitis C co-infection is also associated with increased CD8 T cell activation when compared with HIV mono-infected patients despite ART [104,105].

#### ***Microbial translocation***

Microbial translocation due to impaired mucosal barrier integrity may be another cause of immune activation in HIV infection. Rhesus macaques with chronic SIV infection have disruptions of the epithelial barrier of the colon and increased lipopolysaccharide (LPS) staining. The levels of LPS staining in the colon also correlated with the levels of LPS in the draining lymph nodes and remote peripheral lymph nodes [106]. African green monkeys are natural hosts of SIV. In chronic SIV infection, they display little immune activation, maintain mucosal barrier integrity and do not progress to AIDS [107]. However, the injection of LPS into SIV-infected African green monkeys was associated with increases in T cell activation, sCD14 and SIV viraemia [108]. In HIV-infected patients, plasma LPS also correlated with plasma interferon (IFN)-α and T cell activation levels [109]. These data suggest that impaired mucosal barrier integrity and microbial translocation may induce immune activation.

Markers of microbial translocation e.g. LPS, sCD14 [110] and bacterial 16 s rDNA [111] do not always normalise with ART. LPS can induce tissue factor expression on monocytes [112]. Tissue factor is the initiator of the coagulation cascade [113] and its expression on monocytes is correlated with D-dimer levels [112]; suggesting that microbial translocation may contribute to atherogenesis and increased CVD [112].

### Antiretroviral therapy

Finally, adverse effects of ART may also contribute to SNAEs. A detail review of ART-related toxicities is beyond the scope of this review. Protease inhibitors as a class, as well as indinavir, lopinavir and abacavir may be associated with increased risk of CVD [25,114,115].

Some studies found that the rate of liver-related deaths is increased per year of ART [116,117]. The cohort described by Weber et al have high rates of HBV and HCV infection, 17% and 66%, respectively [116]. Though patients with HBV or HCV infection have about 5-fold greater risk of hepatotoxicity after ART initiation [118-120], ART is essential as it slows fibrosis progression [121] and reduces liver-related mortality by about 10-fold [122]. In patients without chronic viral hepatitis, ART toxicity rarely causes liver-related mortality, at a rate of 0.04 per 1000 person years [123].

ART has been associated with improved renal function and a slower rate of eGFR decline in HIV-infected patients [124,125]. Though tenofovir use is associated with higher risk of acute renal injury and greater loss of renal function than other ART regimen, the overall risk of serious renal event is not high, in 0.5% of patients [126].

## Interventions to reduce SNAEs

Strategies to reduce SNAEs include preventing and reversing immunodeficiency, the modification of traditional risk factors, treatment of co-infections and addressing drivers of immune activation. A wide variety of agents are currently being examined for their potential effects in reducing immune activation and SNAEs (Table 2). However, the majority of studies that have been performed are small, are heterogeneous in terms of ART status and show conflicting findings. The majority of studies used markers of immune activation, in particular CD8 T cell activation as outcome measures. Some recent studies have also included sCD14 and D-dimer. However, randomized placebo-controlled trials that use clinical outcome measures are rare [127].

**Table 2:** Potential strategies to reduce SNAEs

**Potential strategies to reduce SNAEs**	**Interventions investigated or under evaluation**	**References**
**Preventing immunodeficiency**	Initiate ART prior to advanced immunodeficiency	[14,128-134]
**Increasing CD4 T cell recovery**		
Cytokine therapy	Subcutaneous IL-2	[127]
	Subcutaneous IL-7	[137,138]
Modulating lymphoid tissue fibrosis	Pirfenidone	Human data pending
	Angiotensin receptor antagonist	Human data pending
	ACE inhibitor	Human data pending
**Managing co-morbidities**	Smoking cessation	[68,145]
	Optimise blood pressure, lipids and diabetic control	[146,147]
	ART switch	[152-160]
**Reducing chronic antigen stimulation**		
Residual viraemia	Raltegravir intensification	[161-170]
	Maraviroc intensification	[171-174]
HBV and HCV co-infection	Hepatitis B and C treatment	[105,175,176]
CMV co-infection	Valganciclovir	[178]
HSV co-infection	Valacyclovir	[179]
**Reducing inflammation**	Statins	[182-187]
	COX-2 inhibitors	[195,196]
	Aspirin	[199]
	Hydroxychloroquine & Chloroquine	[191-193]
	Leflunomide	[200]
	Prednisone	[201-204]
**Reducing microbial translocation**		
Balancing microbiota	Prebiotic, probiotic and synbiotic	[210-213]
Reducing bacterial/endotoxin load	Rifaximin	Human data pending
	Bovine colostrum	[167]
	Sevelamer	Human data pending
Improving mucosal integrity	Lubiprostone	Human data pending
Reducing inflammation in the gut	Mesalamine	Human data pending

### Preventing and reversing immunodeficiency

#### ***ART initiation prior to advanced immunodeficiency***

Data from randomized controlled trials suggest that deferral of ART initiation until CD4 T cell count <250 cells/μL was associated with increased SNAEs, AIDS-related events and mortality [128-130]. Observational studies suggest that ART initiation at CD4 T cell count >350 cells/μL is associated with lower risk of SNAEs [14], AIDS-defining illness or death when compared to deferring ART [131-134]. The benefit of ART initiation at CD4 T cell count >350 cells/μL when compared to deferring until CD4 T cell count ~350 cells/μL is insignificant in some studies when the analysis is restricted to mortality alone [131,134]. Results from the START study (NCT00867048), a multicenter international trial designed to assess the risks and benefits of initiating ART at CD4 T cell count at >500 or <350 cells/μL will likely shed further light on this issue. Nonetheless, given that the majority of HIV-infected patients start ART with CD4 T cell count <250 cells/μL [135,136], earlier HIV diagnosis and initiation of ART before advanced immunodeficiency will likely reduce SNAEs.

#### ***Improving CD4 T cell recovery***

A number of studies have investigated the use of cytokines critical for T cell homeostasis e.g. IL-2 and IL-7 to enhance CD4 T cell recovery. Though subcutaneous IL-2 administration in concert with ART resulted in sustained increase in CD4 T cell count, this did not translate into clinical benefit [127]. Subcutaneous IL-7 administration also leads to increase in CD4 T cells in phase I and II studies [137,138] but clinical outcomes have not yet been assessed. The restoration of TH17 cells and improvement in TH17/T regulatory cell ratio may be especially important given their roles in mucosal immunity [139,140].

#### ***Modulating the effects of lymphoid tissue fibrosis***

Lymphoid tissue fibrosis is associated with poor CD4 T cell restoration after ART initiation [49]. TGF-β is key in the process of lymphoid tissue fibrosis. Pirfenidone can reduce TGF-β production and has anti-fibrotic effects [45,141]. Pirfenidone administered to rhesus macaques prior to SIV infection was associated with reduced lymph node fibrosis and preservation of lymph node CD4 T cells [142]. TNF blockade with adalimumab was also associated with attenuated TGF-β expression, reduced lymph node fibrosis and preserved lymph node architecture in a recent rhesus macaque study [47]. The effect of pirfenidione or adalimumab on lymph node fibrosis in HIV infection has not been studied to date.

The renin-angiotensin pathway is involved in cardiac, renal and liver fibrosis. Binding of angiotensin II to the angiotensin 1 receptor on cardiac fibroblast, hepatic stellate cells or mesangial cells leads to proliferation as well as collagen and TGF-β synthesis [143,144]. Trials on the effect of angiotensin converting enzyme (ACE) inhibitor e.g. lisinopril (ClinicalTrials.gov identifier: NCT01535235), angiotensin II receptor antagonists e.g. losartan (NCT01852942, NCT01529749) and telmisartan (NCT01928927) in modulating lymphoid tissue fibrosis are currently underway.

### Optimizing cardiovascular risk factors

In the D.A.D study, patients who stopped smoking had about 30% reduction in the risk of CVD [145]. Surprisingly, a reduction in mortality was not seen. This may be because patients ceased smoking after the diagnosis of a serious illness and succumb before the benefit of smoking cessation on mortality can be seen [145]. In the Danish HIV cohort, previous smokers had a >1.5-fold reduction in mortality when compared with current smokers. In addition, though previous smokers have higher rates of AIDS-related deaths when compared with never smokers, the incidence of non-AIDS-related death was not different between previous and never smokers [68]. These data suggest that smoking cessation alone would result in significant benefits and should be encouraged.

Modification of other cardiovascular risk factors e.g. treatment of hypertension, dyslipidaemia and optimal glycaemic control in diabetic patients is critical. Suggested target levels have been published [146,147]. Each 10 mmHg reduction in systolic blood pressure and each 38 mg/dL reduction in total cholesterol is associated with a 5 and 20% reduction in risk of CVD, respectively [148]. However, in patients with known hypertension, diabetes or dyslipidaemia meeting indication for treatment, over 40% were not on treatment [149]. Given that a significant proportion of SNAEs are cardiovascular events, more aggressive detection and management of cardiovascular risk factors will likely reduce SNAEs.

ART modification is a potential strategy to reduce cardiovascular risk [150]. A recent review of switch studies have been published [151]. Switching from stavudine to tenofovir was associated with a reduction in total cholesterol and triglycerides and an increase in limb fat [152]. Switching from protease inhibitors to efavirenz or nevirapine was associated with reduction in total cholesterol [153]. However, this switch is not possible in patients with non-nucleoside reverse transcriptase inhibitor resistance. Switching from lopinavir/ritonavir to atazanavir (both boosted with ritonavir 100 mg or unboosted) was associated with reduction of total cholesterol and triglycerides, though greater reductions were seen with unboosted atazanavir [154-156]. Switching to atazanavir was also associated with a reduction in cardiovascular risk score [157]. Though switching from lopinavir/ritonavir to raltegravir was associated with improvement in lipid profile [158,159], no change in endothelial function was detected [160]. The importance of having fully active backbone antiretrovirals was highlighted in the SWITCHMRK study where patients switched to raltegravir had higher rates of virologic failure [158].

### Suppressing chronic antigen stimulation

#### ***Reducing residual viraemia***

Intensification studies have been performed to assess the impact of adding antiretroviral agents to a suppressive ART regimen (as measured by conventional assays). None of the raltegravir intensification studies were able to demonstrate reduction in ultra-sensitive plasma HIV-RNA levels [161-167]. In addition, the majority of studies also found no reduction in markers of T cell [161-164,167] or monocyte activation [167]. However, some studies have noted a reduction in D-dimer levels [168], T cell activation [165,166,169] as well as an early transient increase in 2-LTR circles post raltegravir intensification [168,170], suggesting that residual viraemia was occurring prior to raltegravir intensification and was contributing to immune activation in some patients.

Maraviroc intensification studies have also been performed and yielded conflicting data. Some found reduction in T cell activation [171-173] whilst others found increase in CD4 and CD8 T cell activation both in the peripheral blood and in the rectal mucosa after maraviroc intensification [174]. Hunt et al postulated that the binding of maraviroc to CCR5 prevents the interaction between CCR5 and its natural ligands. Excess CCR5 ligands may then bind to other chemokine receptor such as CCR3 and CCR4 on T cells, leading to T cell activation [174]. Therefore, the beneficial effect of adding antiretroviral agents to an already suppressive ART regimen is uncertain based on currently available data.

#### ***Treating other co-infections***

HCV treatment and suppression of HCV viraemia is associated with reductions in CD4 and CD8 T cell activation [105]. Sustained virologic response is associated with reduced liver-related complications as well as both liver-related and non liver-related mortality in co-infected patients [175,176]. Unfortunately, HCV treatment may be limited by contraindications, adverse events, high costs, and drug interactions. Next generation agents with higher efficacy and better side effect profiles may revolutionise the management of HIV/HCV co-infected patients [177].

Treatment of other persistent viral infection has also been investigated. Eight weeks of valganciclovir in ART-treated, CMV seropositive patients led to a significant reduction of CMV viraemia as well as a reduction in CD8 T cell activation [178]. In a study targeting HSV co-infection using 12 weeks of valacyclovir in ART-treated, HSV-1 and HSV-2 seropositive patients, no change in T cell activation, CRP or IL-6 levels was demonstrated [179].

### Anti-inflammatory agents

Statins are 3-hydroxy-3-methyl-glutaryl-coenzyme A reductase inhibitors. Not only do statins reduce serum cholesterol [180], they may also have anti-inflammatory properties [181]. Statin use is associated with reduced monocyte activation (unpublished data McComsey et al), decline in CRP levels [182] as well as reduced T cell activation [183] in ART-treated and in untreated, HIV-infected patients [184]. A retrospective observational study of ART-treated patients showed that statin use is associated with a 3-fold reduction in mortality [185]. Though not statistically significant, a trend for reduction in SNAEs [186] and mortality [187] has also been seen in other retrospective observational studies.

Hydroxychloroquine (HCQ) and its analogue chloroquine (CQ) have immunomodulatory, anti-inflammatory and anti-HIV properties [188-190]. In patients with uncontrolled viral replication, the use of CQ was associated with reduced CD8 T cell activation [191] whereas the same effect was not seen with HCQ [192]. However a non-randomized study of HCQ in 20 ART-treated patients showed decline in plasma LPS, IL-6 and reduced T cell and monocyte activation [193]. Thus findings are inconclusive.

COX-2 inhibitors inhibit cyclooxygenase type 2, reducing Prostaglandin E2 production, thereby reducing activation of T cells through the cyclic adenosine monophosphate (cAMP) pathway [194]. Studies on COX-2 inhibitors have been small and reduction in T cell activation tended to occur in viraemic patients [195,196]. However, it is important to bear in mind that COX-2 inhibitors are associated with increased cardiovascular risk, via a direct pharmacologic consequence of inhibition of COX-2 [197]. Therefore assessing the utility of COX-2 inhibitors without using clinical outcome measures may be insufficient.

Aspirin is a cornerstone in the secondary prevention of vascular disease [198]. In a pilot study, aspirin use was associated with reduced platelet activation, a decrease in sCD14 in monocytes and reductions in CD38 and HLA-DR on CD4 and CD8 T cells. However, there was no change in IL-6, D-dimer and CRP [199]. An aspirin study with larger number of participants is in development with the AIDS Clinical Trials Group.

Leflunomide is an immunomodulatory agent that is used in the treatment of rheumatoid arthritis. The administration of leflunomide in untreated, HIV-infected patients for 28 days was associated with a decrease in CD8 T cell activation [200].

The use of prednisone in patients with untreated chronic HIV infection was associated with less CD4 T cell depletion, a decline in CD4 T cell activation and stable HIV viral load [201,202]. Prednisone at 0.5 mg/kg/day in ART-treated patients was also associated with a reduction in CD8 T cell activation and TNF levels as well as a transient decrease in IL-6 [203]. However, in another study using prednisone at 40 mg/day, no reduction in CD4 or CD8 T cell activation, plasma IL-6 or TNF levels was found [204]. Furthermore, long-term prednisone use, especially at doses >7.5 mg/day is associated with significant adverse effects such as osteoporosis, impaired glucose tolerance, dyslipidaemia, weight gain, cataract formation and increased risk of infections [205]. Even short courses have been associated with an increased risk of osteonecrosis in HIV-infected patients [206].

### Targeting microbial translocation

Given that HIV infection has been associated with depressed levels of beneficial gut microbiota and elevated levels of pathogenic microbiota [207], a range of prebiotics (selectively fermented ingredients that changes the growth and/or activity of certain gut microflora, resulting in health benefits [208]), probiotics (live microorganisms that when consumed, confer a health benefit [209]) and synbiotics (combinations of pre and probiotics) are under investigation.

A prebiotic oligosaccharide mixture has been associated with improvement in microbiota composition and reduction in sCD14 in untreated HIV-infected patients [210]. A retrospective cohort study on both ART-treated and untreated HIV-infected patients found that probiotic yogurt consumption was associated with a greater increase in CD4 T cell count even after adjustment for ART [211]. A double-blind randomized placebo-controlled trial in 20 untreated HIV-infected patients found reductions in plasma bacterial DNA and IL-6 levels in patients receiving synbiotics [212]. However, a synbiotic agent in ART-treated women found no change in microbial translocation nor immune activation status despite improvement in the levels of probiotic species [213]. Therefore, more randomized controlled clinical trial data are needed to clarify the effects of pre and probiotics in reducing immune activation.

Bovine colostrum contains oligosaccharides, growth factors, immunoglobulins and antimicrobial peptides and has some activity in alleviating HIV-associated diarrhoea in single arm studies [214-216]. However a randomized controlled trial on the addition of bovine colostrum to suppressive ART found no change in CD4 T cell count, markers of microbial translocation nor T cell activation [167].

A number of new agents that target microbial translocation are under evaluation. Rifaximin is a minimally absorbed oral rifamycin antibiotic that has activity against both gram-positive and gram-negative enteric bacteria [217]. It is effective in the treatment of hepatic encephalopathy, by reducing ammonia-producing enteric bacteria [218,219]. There are currently 3 clinical trials of rifaximin in HIV-infected patients (ClinicalTrials.gov identifier: NCT01654939, NCT01866826 and NCT01466595). Lubiprostone is a chloride channel activator that is used in the treatment of constipation [220]. It has been found to enhance recovery of mucosal barrier function in ischaemic porcine colon [221]. A pilot study of lubiprostone in ART treated, virologically suppressed patients with CD4 T cell count <350 cells/μL (NCT01839734) is currently recruiting. Sevelamer is a phosphate binder that is used in patients with end-stage renal failure [222]. It can also bind to endotoxins and reduce CRP, IL-6 and sCD14 in patients on haemodialysis [223,224]. The trial in untreated, HIV-infected patients (NCT01543958) was completed in June 2013 and results are pending. Mesalamine (5-aminosalicylic acid) is an anti-inflammatory agent used in the management of inflammatory bowel disease [225]. A trial using mesalamine in ART treated, virologically suppressed patients with CD4 T cell count <350 cells/μL (NCT01090102) is currently enrolling.

## Conclusions

Despite the use of ART, HIV-infected patients still have higher mortality and morbidity when compared to the general population. SNAEs occur at the rate of about 1-2 per 100 PYFU and are the predominant causes of morbidity and mortality in HIV-infected patients in the ART era. Many factors contribute to the pathogenesis of SNAEs including the direct effect of HIV and associated immunodeficiency, underlying co-morbidities, immune activation and ART toxicities. Though multiple interventions have been investigated or are ongoing, most of the studies are small, of short duration and clinical outcome measures have not been ascertained. The cost required to investigate the effectiveness of an intervention to reduce SNAEs may be prohibitively high as it will require thousands of participants with possibly several years of follow up.

Currently, the interventions with evidence to suggest an association with reduced risk of SNAEs or mortality are starting ART before advanced immunodeficiency, smoking cessation, optimisation of cardiovascular risk factors and treatment of HCV infection. Clinicians should focus their attention on addressing these issues prior to the availability of further data.

## Abbreviations

ACE: Angiotensin converting enzyme; ART: Antiretroviral therapy; cAMP: Cyclic adenosine monophosphate; CDC: Centers for disease control and prevention; CQ: Chloroquine; CRP: C-reactive protein; CVD: Cardiovascular disease; EBV: Epstein-Barr virus; FRC: Fibroblastic reticular cell; HBV: Hepatitis B virus; HCV: Hepatitis C virus; HCQ: Hydroxychloroquine; HPV: Human papillomavirus; IL-7: Interleukin-7; IFN: Interferon; LPS: Lipopolysaccharide; NAEs: Non-AIDS events; PYFU: Person-years of follow-up; sCD14: Soluble CD14; SIR: Standardized incidence ratio; SMART: Strategies for management of antiretroviral therapy; SNAEs: Serious non-AIDS events; START: Strategic timing of antiretroviral treatment; TGF: Transforming growth factor; TNF: Tumour necrosis factor.

## Competing interests

The authors declare that they have no competing interests.

## Authors’ contributions

DH, JA, IS contributed to the writing of the manuscript. All authors read and approved the final manuscript.
